# Generative AI in microbial evolution and resistance: toward robust, explainable, and equitable predictions

**DOI:** 10.3389/fmicb.2025.1705320

**Published:** 2025-12-05

**Authors:** Fahim Sufi

**Affiliations:** COEUS Institute, New Market, VA, United States

**Keywords:** generative artificial intelligence, microbial evolution, antimicrobial resistance, drug discovery, explainability and biosafety, explainable AI, GPT in medical domain

## Abstract

Antimicrobial resistance (AMR) is one of the most urgent challenges in modern microbiology, both an evolutionary inevitability and a global health crisis shaped by clinical practices, ecological disruption, and social inequities. Generative artificial intelligence (AI) and large language models (LLMs) present new opportunities to anticipate resistance pathways, design novel antimicrobial agents, and guide interventions that are informed by evolutionary dynamics. Their successful integration, however, depends on addressing three fundamental imperatives. The first is evolutionary robustness, requiring models that incorporate mutation, horizontal gene transfer, and adaptive landscapes to move beyond retrospective classification toward predictive evolutionary inference. The second is explainability and biosafety, which demand interpretable and biologically credible outputs that clinicians, microbiologists, and policymakers can trust, while safeguarding against dual use risks. The third is data equity, which calls for strategies that mitigate structural biases in global microbial datasets and ensure that predictive systems serve the populations most affected by AMR. This Perspective advances the view that generative AI must be conceived as a transformative epistemic infrastructure that is evolution aware, transparent, and globally inclusive, capable of supporting sustainable drug discovery, adaptive surveillance, and equitable microbiological futures.

## 1 Introduction

Antimicrobial resistance (AMR) has emerged as one of the gravest global health threats, eroding decades of medical progress and exposing the fragility of current therapeutic paradigms. While resistance is an evolutionary inevitability under sustained selective pressures, its contemporary acceleration reflects not only microbial adaptation but also structural factors such as clinical misuse, ecological disruption, and inequities in healthcare infrastructures (Wortel et al., 2023; Pinheiro, 2024). Traditional epidemiological and microbiological models—while foundational—struggle to capture the complex, multiscale interactions that govern resistance emergence and dissemination, leaving critical blind spots in prediction and intervention (Charlebois, 2023; Denk-Lobnig and Wood, 2023).

Generative artificial intelligence (AI), particularly large language models (LLMs) and protein language models, now introduces the possibility of transcending these limitations by simulating unseen microbial interactions, inferring latent evolutionary trajectories, and generating hypotheses for novel antimicrobial discovery (Mulat et al., 2025). However, their deployment in microbiology is far from straightforward. At stake is not merely the technical optimization of predictive accuracy, but the epistemological and ethical legitimacy of these models when applied to life-critical domains (Khosravi et al., 2025). Without careful integration, generative systems risk becoming biologically naïve, clinically opaque, or inequitable in their global relevance. In healthcare more broadly, AI has already been applied in diagnostic imaging, sepsis detection, and predictive patient monitoring, illustrating both the transformative potential and the pitfalls of clinical AI. These experiences underscore the importance of transparency, validation, and bias mitigation, principles that should equally guide AI deployment in microbiology.

This Perspective frames the debate around three interlocking imperatives that must shape the integration of generative AI into microbiological research. First, evolutionary robustness: can AI architectures be systematically aligned with the principles of microbial evolution—mutation rates, horizontal gene transfer, adaptive landscapes—to anticipate resistance rather than retroactively describe it (Temsah and Al-Tawfiq, 2025; Petrungaro et al., 2021)? Second, explainability and biosafety: how can black-box models be rendered interpretable and trustworthy for microbiologists, clinicians, and policymakers, while simultaneously addressing dual-use concerns (Yin et al., 2025; Mediouni et al., 2025; Wysocka et al., 2024)? Third, data equity: in what ways do entrenched biases in global microbial datasets skew predictions, and how can fairness-aware approaches mitigate inequities across underrepresented pathogens and geographies (Tripathi et al., 2024; Mohammed Aarif et al., 2025; Prosperi et al., 2022)?

By situating these imperatives at the center of inquiry, this article advances the argument that generative AI must be conceived not as a neutral computational engine but as a transformative epistemic infrastructure. Only by embedding evolutionary science, interpretability, and equity into its foundations can AI contribute to sustainable antimicrobial discovery, robust surveillance, and globally just microbiological futures.

## 2 Evolutionary robustness: embedding generative AI in microbial dynamics

The predictive value of AI in microbiology hinges on its ability to model evolutionary processes rather than merely extrapolate from existing datasets. Antimicrobial resistance emerges not as a static attribute but as a dynamic trajectory, shaped by mutations, horizontal gene transfer, and fluctuating ecological pressures. Generative AI offers the potential to simulate these processes, but unless evolutionary dynamics are explicitly embedded, its outputs risk remaining biologically naïve.

Eco-evolutionary simulations have already demonstrated how sensitive strains can re-establish themselves under favorable ecological conditions, thereby suppressing resistant populations (Temsah and Al-Tawfiq, 2025). Generative architectures could extend this principle by testing counterfactuals—asking not only “what is” but “what could be”—thus enabling anticipatory strategies for infection control. Yet this requires moving beyond correlation-driven models to those explicitly parameterized by ecological and evolutionary laws.

Systems biology provides another critical entry point. By linking molecular perturbations to community-level outcomes, multiscale models aim to predict the repeatability of evolutionary trajectories (Charlebois, 2023; Denk-Lobnig and Wood, 2023). However, their predictive power is often undermined by incomplete data and context-dependence. Generative AI could help bridge these gaps by inferring hidden variables from noisy datasets, but this raises an epistemic risk: if models hallucinate plausible but biologically unfounded patterns, they may generate elegant predictions that collapse under empirical scrutiny. Rigorous iterative validation through experimental evolution remains indispensable (Petrungaro et al., 2021).

Genome-scale metabolic and growth models represent one of the most promising arenas for integration. These models capture how metabolic fluxes and physiological constraints interact with mutational processes to yield adaptive resistance (Wortel et al., 2023; Pinheiro, 2024). Generative AI could expand their reach by proposing novel mutational pathways and simulating evolutionary “shortcuts” that microbes may exploit. However, unless such proposals are tested against real-world phenotypic outcomes, they risk reinforcing a speculative layer of modeling divorced from clinical utility. Recent advances in diffusion-based protein language models and generative adversarial networks have demonstrated capacity to design antimicrobial peptides *de novo*, grounding the discussion of drug discovery in concrete algorithmic advances.

Finally, real-time AI surveillance highlights the potential of adaptive feedback loops. Machine learning tools trained on genomic data can already predict resistance phenotypes in clinical isolates (Wang et al., 2022) and track temporal shifts in microbial behavior (Pourhajibagher et al., 2024). Embedding generative simulations within such pipelines could allow clinicians to dynamically adjust therapies before resistance becomes entrenched. Yet here again lies a tension: the speed of generative prediction may outpace the slow rhythms of experimental confirmation, raising the risk of premature clinical application.

In sum, evolutionary robustness demands more than algorithmic sophistication. It requires a principled synthesis of eco-evolutionary theory, systems biology, and empirical validation. Generative AI will only be transformative if it ceases to function as a retrospective classifier and instead becomes an engine of evolutionary inference—capable of anticipating the microbial futures we most need to avoid.

## 3 Explainability, trust, and biosafety

The opacity of LLM-driven predictions constitutes not only a technical limitation but an epistemic risk for microbiology. Without transparency, such models cannot be meaningfully interrogated, reproduced, or trusted in clinical contexts where decisions have life-and-death consequences. Attention visualization and interpretable feature extraction represent one pathway toward enhanced transparency, offering microbiologists and clinicians the ability to inspect which genomic or phenotypic features drive resistance predictions (Yin et al., 2025). Yet these mechanisms must go beyond visualization; they must demonstrate biological plausibility by linking genotypic markers directly to phenotypic resistance outcomes (Mediouni et al., 2025). Methods such as SHAP (SHapley Additive Explanations) have already been used in genomic prediction models to highlight the contribution of specific mutations to resistance phenotypes, providing microbiologists with transparent, biologically grounded explanations.

Factuality frameworks are especially critical in generative contexts, where the risk of plausible but false outputs is amplified. Systematic evaluation of factual accuracy has been proposed as a safeguard against such spurious results, ensuring that AI-generated insights are not only internally consistent but also biologically credible (Wysocka et al., 2024). At the policy level, indicator-based models that contextualize resistance within broader ecological and societal determinants provide additional interpretability, aligning predictions with public health decision-making priorities (Monaco et al., 2025).

Hybrid systems offer another layer of reliability. By combining rule-based inference with machine learning, these models embed established biological knowledge into the predictive process, reducing the risks of black-box decision-making and increasing clinical confidence (Gil-Gil et al., 2021). However, transparency cannot stop at the algorithmic level: open disclosure of training data quality and adherence to regulatory standards are indispensable for safeguarding against dual-use risks, including the potential misuse of AI systems to design resistant strains (Giacobbe et al., 2024).

Finally, the integration of multiomics data with protein language models extends explainability to a universal scale. Such models not only close annotation gaps but also provide a coherent framework for resistance gene classification across diverse microbial contexts, supporting a more interpretable and systematic mapping of the resistome (Yadalam et al., 2025).

Taken together, explainability must be treated as a constitutive property of AI in microbiology, not an optional afterthought. A system that produces accurate yet uninterpretable predictions risks undermining both clinical adoption and public trust. Black-box models in this domain are therefore not merely suboptimal—they are epistemically irresponsible.

## 4 Data bias and global inequity in microbial predictions

Bias pervades microbial datasets and systematically undermines the reliability of AI-driven resistance prediction. These biases are not incidental but structural, reflecting historical inequities in how microbial data are collected, curated, and shared. Underrepresentation of pathogens from low-resource geographies leads to skewed predictions, leaving precisely those populations most vulnerable to antimicrobial resistance underserved by global models (Tripathi et al., 2024; Mohammed Aarif et al., 2025; Prosperi et al., 2022). Overrepresentation of clinically prioritized pathogens from high-income regions exacerbates this imbalance, producing models that appear robust in benchmark testing yet fail in diverse clinical realities.

Generative models carry a dual risk in this context: they inherit biases from the training data, and they amplify them by generating outputs that reinforce existing data distributions (Khosravi et al., 2025). In the microbial domain, where strain diversity is vast and ecological contexts are heterogeneous, such amplification can be particularly damaging. As Mulat et al. (2025) note, narrow datasets undermine generalizability, producing brittle predictions that collapse when confronted with underrepresented microbial ecologies. Similarly, population stratification and the co-occurrence of resistance markers distort machine learning signals, inflating performance metrics while masking underlying fragility (Libiseller-Egger et al., 2020).

Mitigating these inequities requires a multipronged approach. Fairness-aware data augmentation and adversarial debiasing can expand representational diversity within training datasets, while transfer learning enables the adaptation of robust models to rare or under-sampled pathogens (Khosravi et al., 2025; Mohammed Aarif et al., 2025). Causally informed frameworks provide an even stronger safeguard by explicitly modeling bias propagation and identifying structural confounders (Prosperi et al., 2022). Moreover, the incorporation of phylogenetic relationships as structural priors constrains generative outputs, ensuring that predictions are consistent with evolutionary trajectories rather than spurious correlations (Yurtseven et al., 2023).

Crucially, equity must be more than a technical correction; it must be a measurable and auditable property of microbial AI systems. Without mechanisms for transparency, accountability, and global data-sharing, generative AI risks entrenching what may be termed a form of “microbial colonialism”—a future where predictive infrastructures work best for the Global North while systematically neglecting the microbial realities of the Global South.

## 5 Conceptual synthesis: paradigm shifts required

**Figure 1:**
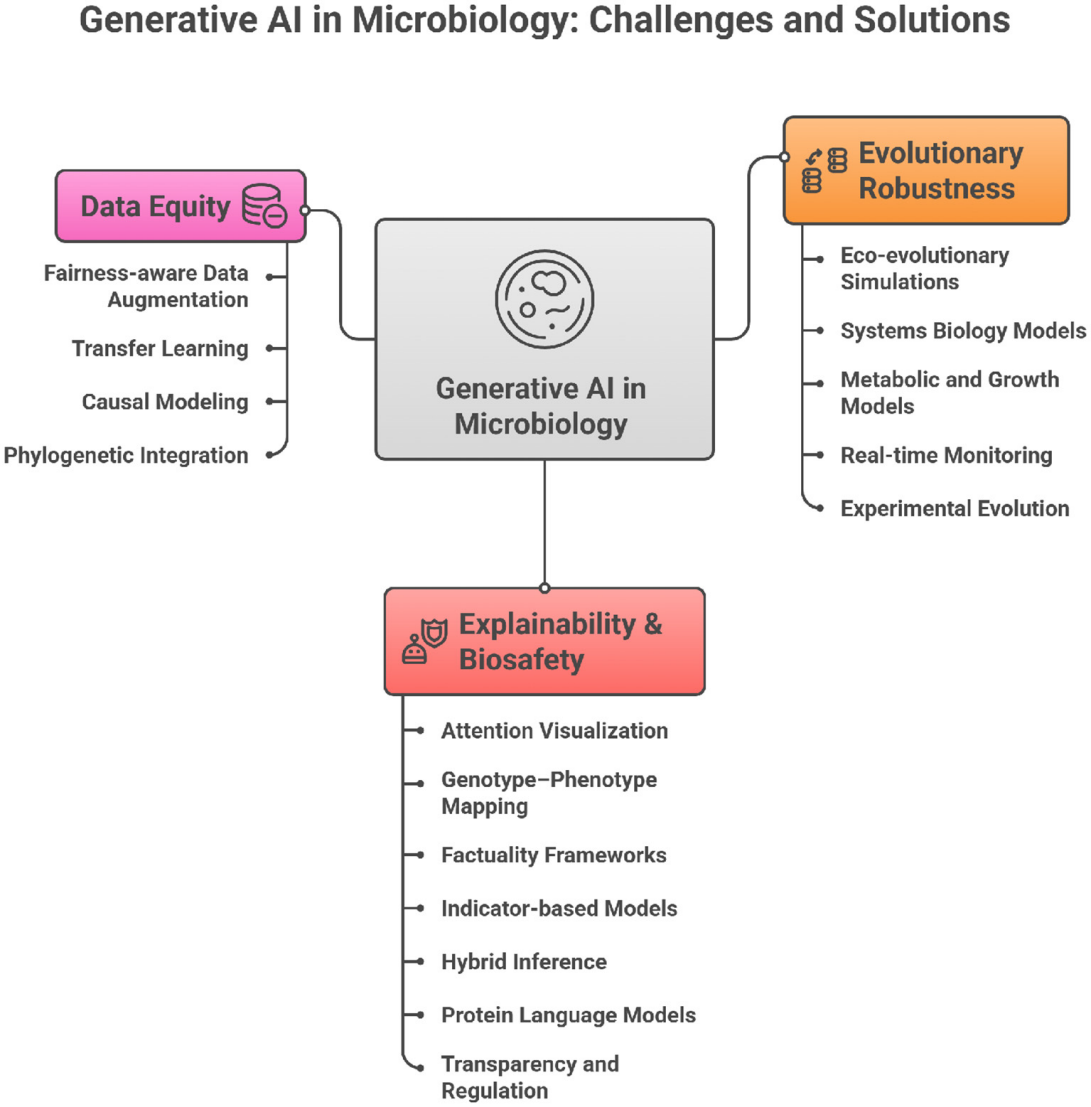
Conceptual framework of generative AI in microbiology showing the three core imperatives (i.e., evolutionary robustness, explainability and biosafety, and data equity).

The integration of generative AI into microbiology is not merely a technical challenge but a conceptual one. Current practices in AMR prediction often prioritize performance benchmarks over biological grounding, interpretability, or equity. To transform AI into a responsible partner in microbial science, paradigm shifts are required across five dimensions: evolutionary logic, explainability, data equity, validation, and biosafety. Table 1 summarizes these contrasts, while Figure 1 provides a visual framework linking the three central imperatives—evolutionary robustness, explainability & biosafety, and data equity—to their corresponding methodological strategies.

**Table 1:** From current practices to required paradigm shifts in AI-driven microbiology.

**Dimension**	**Current practice (limitations)**	**Required paradigm shift (future direction)**
Evolutionary logic	Static predictions decoupled from evolutionary processes (Wortel et al., 2023; Pinheiro, 2024)	Explicit embedding of mutation rates, gene transfer, and fitness landscapes (Temsah and Al-Tawfiq, 2025; Charlebois, 2023; Denk-Lobnig and Wood, 2023)
Explainability	Black-box predictions with limited interpretability (Yin et al., 2025)	Transparent, biologically grounded, policy-relevant interpretability (Mediouni et al., 2025; Wysocka et al., 2024; Monaco et al., 2025)
Data equity	Bias toward well-studied pathogens and geographies (Tripathi et al., 2024; Prosperi et al., 2022)	Fairness-aware, causally informed, globally representative models (Mohammed Aarif et al., 2025; Khosravi et al., 2025; Yurtseven et al., 2023)
Validation	One-off benchmarks disconnected from empirical feedback (Petrungaro et al., 2021)	Iterative validation via experimental evolution and real-world surveillance (Denk-Lobnig and Wood, 2023)
Biosafety	Minimal attention to dual-use or misuse risks (Giacobbe et al., 2024)	Integration of biosafety and regulatory compliance at design stage (Gil-Gil et al., 2021; Yadalam et al., 2025)

## 6 Outlook

Generative AI is not a panacea for antimicrobial resistance but a new epistemic instrument. Its promise hinges on three imperatives: embedding evolutionary dynamics into predictive architectures, designing interpretability as a constitutive principle, and enforcing fairness in data representation. Failure to do so risks constructing an AI infrastructure that accelerates inequity and mistrust.

Conversely, if these imperatives are realized, generative AI could inaugurate a new era of microbiological science—one that is predictive, transparent, and globally equitable. The future of antimicrobial resistance research will depend less on the size of our models than on the depth of our epistemic commitments.

## Data Availability

The original contributions presented in the study are included in the article/supplementary material, further inquiries can be directed to the corresponding author.
